# Cardiac sarcoidosis: A long term follow up study

**DOI:** 10.1371/journal.pone.0238391

**Published:** 2020-09-18

**Authors:** Patrice Cacoub, Catherine Chapelon-Abric, Matthieu Resche-Rigon, David Saadoun, Anne Claire Desbois, Lucie Biard

**Affiliations:** 1 UPMC Univ Paris 06, UMR 7211, and Inflammation-Immunopathology-Biotherapy Department (DHU i2B), Sorbonne Universités, Paris, France; 2 INSERM, UMR_S 959, Paris, France; 3 CNRS, FRE3632, Paris, France; 4 Department of Internal Medicine and Clinical Immunology, AP-HP, Groupe Hospitalier Pitié-Salpêtrière, Paris, France; 5 Department of Biostatistics and Medical Information, AP-HP Hôpital Saint Louis, Paris, France; 6 INSERM, UMRS 1153, ECSTRRA Team, Paris, France; 7 Paris Diderot, Paris 7 University, Paris, France; Ospedale del Cuore G Pasquinucci Fondazione Toscana Gabriele Monasterio di Massa, ITALY

## Abstract

**Background:**

Prognostic factors are lacking in cardiac sarcoidosis (CS), and the effects of immunosuppressive treatments are unclear.

**Objectives:**

To identify prognostic factors and to assess the effects of immunosuppressive drugs on relapse risk in patients presenting with CS.

**Methods:**

From a cohort of 157 patients with CS with a median follow-up of 7 years, we analysed all cardiac and extra-cardiac data and treatments, and assessed relapse-free and overall survival.

**Results:**

The 10-year survival rate was 90% (95% CI, 84–96). Baseline factors associated with mortality were the presence of high degree atrioventricular block (HR, 5.56, 95% CI 1.7–18.2, *p* = 0.005), left ventricular ejection fraction below 40% (HR, 4.88, 95% CI 1.26–18.9, *p* = 0.022), hypertension (HR, 4.79, 95% CI 1.06–21.7, *p* = 0.042), abnormal pulmonary function test (HR, 3.27, 95% CI 1.07–10.0, *p* = 0.038), areas of late gadolinium enhancement on cardiac magnetic resonance (HR, 2.26, 95% CI 0.25–20.4, *p* = 0.003), and older age (HR per 10 years 1.69, 95% CI 1.13–2.52, *p* = 0.01). The 10-year relapse-free survival rate for cardiac relapses was 53% (95% CI, 44–63). Baseline factors that were independently associated with cardiac relapse were kidney involvement (HR, 3.35, 95% CI 1.39–8.07, *p* = 0.007), wall motion abnormalities (HR, 2.30, 95% CI 1.22–4.32, *p* = 0.010), and left heart failure (HR 2.23, 95% CI 1.12–4.45, *p* = 0.023). After adjustment for cardiac involvement severity, treatment with intravenous cyclophosphamide was associated with a lower risk of cardiac relapse (HR 0.16, 95% CI 0.033–0.78, *p* = 0.024).

**Conclusions:**

Our study identifies putative factors affecting morbidity and mortality in cardiac sarcoidosis patients. Intravenous cyclophosphamide is associated with lower relapse rates.

## Introduction

Sarcoidosis is a multi-system granulomatous disease of unknown origin with an overall prevalence from 10 to 20 per 100,000 in white American and European patients to 35 per 100,000 in African American patients [1–3]. Clinically manifest cardiac involvement—known as cardiac sarcoidosis (CS)—occurs in 5% to 11% [4–6] whereas cardiac involvement was found in 25% of patients with sarcoidosis on autopsies [7, 8]. Such findings are consistent with data using late gadolinium enhancement on cardiac magnetic resonance imaging (MRI) [9, 10]. Between 16% and 35% of patients presenting with complete atrioventricular block [11, 12] or ventricular tachycardia of unknown etiology [12–14] have previously undiagnosed CS. Core left ventricular biopsies at the time of left ventricular assist device implantation found undiagnosed CS in 3.4% of patients [15], and 3% of explanted hearts had undiagnosed CS [16]. Congestive heart failure is a common presenting feature, as is sudden death. More rarely, CS has been associated with atrial arrhythmias and valvulopathy, coronary vasculitis, acute myocarditis, and arrhythmogenic right ventricular cardiomyopathy [17].

Cardiac involvement has been reported to account for 25% of all deaths from sarcoidosis in the United States and 85% in Japanese series [7]. There is controversy as to the prognosis of patients with clinically silent CS. In patients with clinically manifest disease, the extent of left ventricle dysfunction has been reported as a predictor of survival [18–24]. Despite the lack of randomized controlled trials, the use of moderate to high dose glucocorticosteroids is widely accepted [23–28], with the highest quality data related to atrioventricular block [18], left ventricular dysfunction and ventricular arrhythmias [6, 25–27]. Immunosuppressant are used as a second-line agent in refractory cases of CS and/or if there are significant steroid side effects [4, 29].

In this retrospective study of a large cohort of CS patients with a long follow up, we aimed to: 1) identify baseline prognostic factors influencing overall survival and relapses; and 2) assess the effects of immunosuppressive drugs on relapse risk.

## Methods

### Patients

Data from 690 patients with systemic sarcoidosis diagnosed and followed in a single national referral centre at La Pitié-Salpêtrière University Hospital, Paris, France, between January 1980 and February 2016 were collected. All patients who met the World Association for Sarcoidosis and Other Granulomatous Disorders (WASOG) criteria for cardiac sarcoidosis [28, 29] and whose cardiac symptoms had appeared in 1980 or later were selected. Even in the presence of suggestive manifestations, cardiac biopsy is rarely realized because of its own risk and its poor diagnostic performance. In the present series, 5 out of 157 patients had had a cardiac biopsy with typical pathological features of sarcoidosis found in 3 of them. Of note, as for the present study we aimed to analyse the effect of steroid or immunosuppressant therapy, we did not include sarcoidosis patients who presented only the criteria “steroid ± immunosuppressant-responsive cardiomyopathy or heart block” [28, 29].

Any new cardiac (e.g. dyspnoea, syncope, heart failure, troubles of cardiac rhythm or conduction…) or non-cardiac symptoms attributed to sarcoidosis by the patient’s referral physician defined a relapse [5]. When appropriate, the relapse was confirmed by either radiological (echocardiography, cardiac MRI, cardiac FDG-PET scan, brain and/or spine MRI etc…) or pathological evidence. Ventricular extrasystoles were considered as a cardiac sarcoidosis manifestation when > 1000/24 hours. Hypertension was defined as either diastolic blood pressure > 90 mmHg or systolic blood pressure > 140 mmHg. Whenever a biopsy was performed, a relapse was confirmed if the histopathological analysis revealed a well-defined non-caseating granuloma. Outcomes were assessed by the vital and relapse-free survivals. Patients with one or more-than-one relapse were considered as relapsers.

A switch or an adjustment of the dose of immunosuppressant was done within the following ranges: methotrexate 0.3–0.4 mg/kg/week; mycophenolic acid 2.0 to 3.0 g/day; azathioprine 50 to 150 mg/day. Intravenous cyclophosphamide was administered at the dose of 1g monthly, and infliximab at 5 mg/kg at 0, 2, 6 and then every 8 weeks.

The institutional review board of the Assistance Publique-Hôpitaux de Paris approved this observational retrospective study, and informed consent was not required.

Patient and Public Involvement: it was not appropriate or possible to involve patients or the public in the design, or conduct, or reporting, or dissemination plans of our research.

### Statistical analyses

Continuous variables are presented with the median and interquartile range (IQR); categorical variables are presented with counts and proportions.

The date of the CS diagnosis was considered to be either the sarcoidosis diagnosis date (if the cardiac signs occurred concomitant with or prior to the sarcoidosis diagnosis), or the date of the first cardiac signs. Overall (OS) and relapse-free survival (RFS), as defined previously [5], were estimated using the Kaplan-Meier method. Survival functions were compared using the log rank test. Univariate analyses were performed in Cox regression models to identify baseline factors associated with OS and RFS. For RFS and cardiac-RFS analyses, multivariate models were selected by backward stepwise selection on p-values, using variables that were significant at a 5% level in univariate analysis. The association between recurrent relapse (any localization and cardiac) and sequences of CS treatments was examined in the subgroup of patients with at least one clinical Birnie’s criterion other than therapeutic response (see methods), using the Andersen-Gill Cox approach; this accounted for potential intra-patient correlation across observations. These recurrent events analyses were adjusted for New York Heart Association (NYHA) status (class 3–4 vs. 1–2), presence of cardiac rhythm disorders (yes vs. no), and presence of atrioventricular or ventricular conduction abnormalities (yes vs. no) during the follow-up.

All tests were two-sided, and a p-value below 0.05 was considered significant. Analyses were performed using R statistical platform software, version 3.2.2.

### Patient and public involvement in research

We acknowledge that patient and public involvement is of importance. However, this appears not appropriate for the present papers.

## Results

### Characteristics of cardiac sarcoidosis patients

One hundred and seven patients [92 (59%) men, 77 (50%) Caucasians] met the new WASOG criteria for CS (median age 40 years, IQR 32–49) [29], with a median follow-up of 7 years (6 months– 32 years), 1 to 16 follow up visits, and a 60 months follow up in 67%. The cardiac signs occurred either prior to [n = 15, 10%], concomitant with [n = 54, 34%] or after [n = 88, 56%] the sarcoidosis diagnosis.

The main demographic data and extra-cardiac features are summarized in **Table 1**. Constitutional symptoms were observed in 43% of CS patients and 135/157 (86%) patients had two or more extra-cardiac sites, including mediastinal lymph nodes and/or lungs (89%), nervous system (42%), skin (31%), peripheral lymph nodes (30%), eyes (29%), and joints (24%). Elevated serum angiotensin-converting enzyme was noted in 86 (55%) patients.

**Table 1:** Main extra-cardiac features of 157 cardiac sarcoidosis patients.

Variables		Number (%) or Median (IQR)
**General features**		
Age at cardiac sarcoidosis diagnosis (yrs)		40 (32; 49)
Male gender		92/157 (59)
Ethnic background		
	Caucasian	77 (50)
	African / Caribbean	43 (28)
	North African	34 (22)
	Other	3 (2)
Active smoking habit		20 (13)
**Extra-cardiac involvement**		
Number of extra-cardiac sites		
	0	2 (1)
	1	20 (13)
	2	45 (29)
	3	39 (25)
	> 3	51 (32)
Abnormal chest X-ray		130/146 (89%)
Class 0		16 (11)
Class I		38 (26)
Class II		67 (46)
Class III		25 (17)
General symptoms		67 (43)
Skin		48 (31)
Lymph node		47 (30)
Central nervous system		45 (29)
Eye		45 (29)
Joints		37 (24)
Liver or spleen		36 (23)
Exocrine gland		27 (17)
Ear, nose and throat		8 (5)
Kidney		8 (5)
Peripheral nervous system		5 (3)
Bones		4 (3)
Digestive tract		3 (2)

The main clinical cardiac features are detailed in **Table 2.** Clinical manifestations of heart involvement were noted in all 157 (100%) patients, including ventricular block in 48/157 (31%), atrioventricular block in 27/157 (17%), ventricular arrhythmia in 27/157 (17%), left heart failure in 15/157 (10%), syncope in 10/157 (6%), and class 3 or 4 NYHA dyspnoea in 10/157 (6%). Of note, similar rates of NYHA class of dyspnoea were present whatever the results of pulmonary function tests, suggesting that most dyspnoea were related to cardiac dysfunction (**S1 Table**).

**Table 2:** Main clinical cardiac features of 157 cardiac sarcoidosis patients, according to the presence of cardiac relapse.

Variables	Total	Cardiac relapse	No cardiac relapse
**Total number of patients**	157	63	94
**CLINICAL MANIFESTATIONS, number (%)**			
*Palpitation*	20 (13)	8 (13)	12 (13)
*Syncope*	10 (6)	4 (6)	6 (6)
*NYHA class dyspnoea*			
*1*	119 (76)	44 (70)	75 (80)
*2*	28 (18)	15 (24)	13 (14)
*3*	7 (4)	3 (5)	4 (4)
*4*	3 (2)	1 (2)	2 (2)
*Left heart failure*	15 (10)	10 (16)	5 (5)
*Right heart failure*	3 (2)	3 (5)	0 (0)
**ELECTROCARDIOGRAM, number (%)**			
*Any abnormality*	109 (69)	46 (73)	63 (67)
**Atrial dysfonction**	55 (35)	20 (32)	35 (37)
*Sinusal tachycardia*	49 (31)*	17 (27)	32 (34)
*Fibrillation or flutter*	9 (6)	3 (5)	6 (6)
**Ventricular arrhythmia**	27 (17)*	9 (14)	18 (19)
*Ventricular extrasystoles*	21 (13)	8 (13)	13 (14)
*Ventricular tachycardia*	13 (8)	4 (6)	9 (10)
**Atrioventricular block**	27 (17)*	16 (25)	11 (12)
*1*^*st*^ *degree*	15 (10)	7 (11)	8 (9)
*2*^*nd*^ *degree*	9 (6)	6 (10)	3 (3)
*3*^*rd*^ *degree*	6 (4)	5 (8)	1 (1)
**Ventricular block**	38 (24)*	13 (21)	25 (27)
*Right bundle branch*	33 (21)	11 (17)	22 (23)
*Left bundle branch*	4 (3)	2 (3)	2 (2)
*Abnormal axis deviation*	35 (22)	14 (22)	21 (22)
**Left ventricular hypertrophy**	7 (4)	2 (3)	5 (5)
**Q wave/ST-T changes**	5 (3)	3 (5)	2 (2)

* 3 patients had both sinus tachycardia and atrial fibrillation/flutter; 7 patients had both ventricular extrasystoles and tachycardia; 3 patients had a 1^st^ degree and a 2^nd^ degree atrioventricular block; and 1 patient had left bundle branch block.

**Table 3** summarizes the main cardiac imaging results. Echocardiography found abnormalities in 98/157 (62%) patients, including wall motion abnormalities in 20/157 (13%), thick interventricular septum in 18/156 (12%), and LVEF below forty percent in 15/152 (10%). Cardiac thallium scintigraphy showed localized or diffuse perfusion defects in a pattern consistent with CS in 107/133 (80%). Cardiac MRI was abnormal in 68/91 (75%) patients including early 12/88 (14%) or late 39/88 (44%) gadolinium enhancement, and low LVEF in 28/88 (32%). Cardiac FDG PET scan showed a patchy uptake in 12/37 (32%).

**Table 3:** Main imaging cardiac features of 157 cardiac sarcoidosis patients, according to the presence of cardiac relapse.

Variables	Total	Cardiac relapse	No cardiac relapse
**Total number of patients**	157	63	94
**ECHOCARDIOGRAPHY, (n = 157), number (%)**			
*Any abnormality*	98 (62)	48 (76)	50 (53)
*Diffuse hypokinesia*	41 (26)	23 (37)	18 (19)
*Localized hypokinesia*	40 (25)	20 (32)	20 (21)
*Wall motion abnormalities*	20 (13)	13 (21)	7 (7)
*Thick interventricular septum*	18 (12)	6 (10)	12 (13)
*Abnormal pericardium*	18 (11)	6 (10)	12 (13)
*Left ventricular ejection fraction*			
*> 50%*	112 (74)	41 (69)	71 (76)
*50–40%*	25 (16)	10 (17)	15 (16)
*< 40%*	15 (10)	8 (14)	7 (8)
**CARDIAC SCINTIGRAPHY (n = 133), number (%)**			
*Localized perfusion defects*	98 (74)	40 (73)	58 (74)
*Diffuse perfusion defects*	9 (7)	5 (9)	4 (5)
**CARDIAC MRI (n = 91), number (%)**			
*Any abnormality*	68 (75)	28 (85)	40 (69)
Hypersignals (T1 mapping)*	24 (28)	11 (34)	13 (24)
*Early gadolinium enhancement* †	12 (14)	6 (18)	6 (11)
*Delayed gadolinium enhancement* †	39 (44)	19 (58)	20 (36)
*Localized hypokinesia*†	7 (8)	3 (9)	4 (7)
*Low left ventricular ejection fraction*†	28 (32)	11 (33)	17 (31)
Abnormal pericardium**	9 (11)	1 (3)	8 (15)
**CARDIAC PET SCAN (n = 37), number (%)**			
*Patchy uptake*	12 (32)	2 (13)	10 (45)

MRI, magnetic resonance imaging; PET scan, positron emission tomography.

*n = 87

†: n = 88

**n = 85

Patients were given steroids either alone (n = 92) or in association with immunosuppressive drugs [n = 120, including intravenous cyclophosphamide (n = 79), methotrexate (n = 59), mycophenolic acid (n = 45), hydroxychloroquine (n = 29), infliximab (n = 14) and azathioprine (n = 8)] (**S2 Table**). The median (Q1-Q3) daily dose of steroids at entry and the end of follow-up was 53 mg (30–75) and 5 mg (3–10), respectively. Main steroids-related adverse effects were hypertension (24/157, 15%), diabetes (19/157, 12%), obesity (15/157, 11%), infections (13/157, 8%), osteoporosis (9/157, 6%), and tuberculosis (1/157, <1%). All patients also received conventional cardiac treatments, i.e. diuretics, ACE inhibitors, beta-blockers, anti-arrhythmic drugs, etc. Other cardiac treatments included a pace maker (7 patients), an implantable cardioverter defibrillator (2 patients), a pace maker plus an implantable cardioverter defibrillator (2 patients), a radio-ablation (3 patients), and a heart transplantation (2 patients).

### Prognostic factors

#### Survival

**Fig 1:**
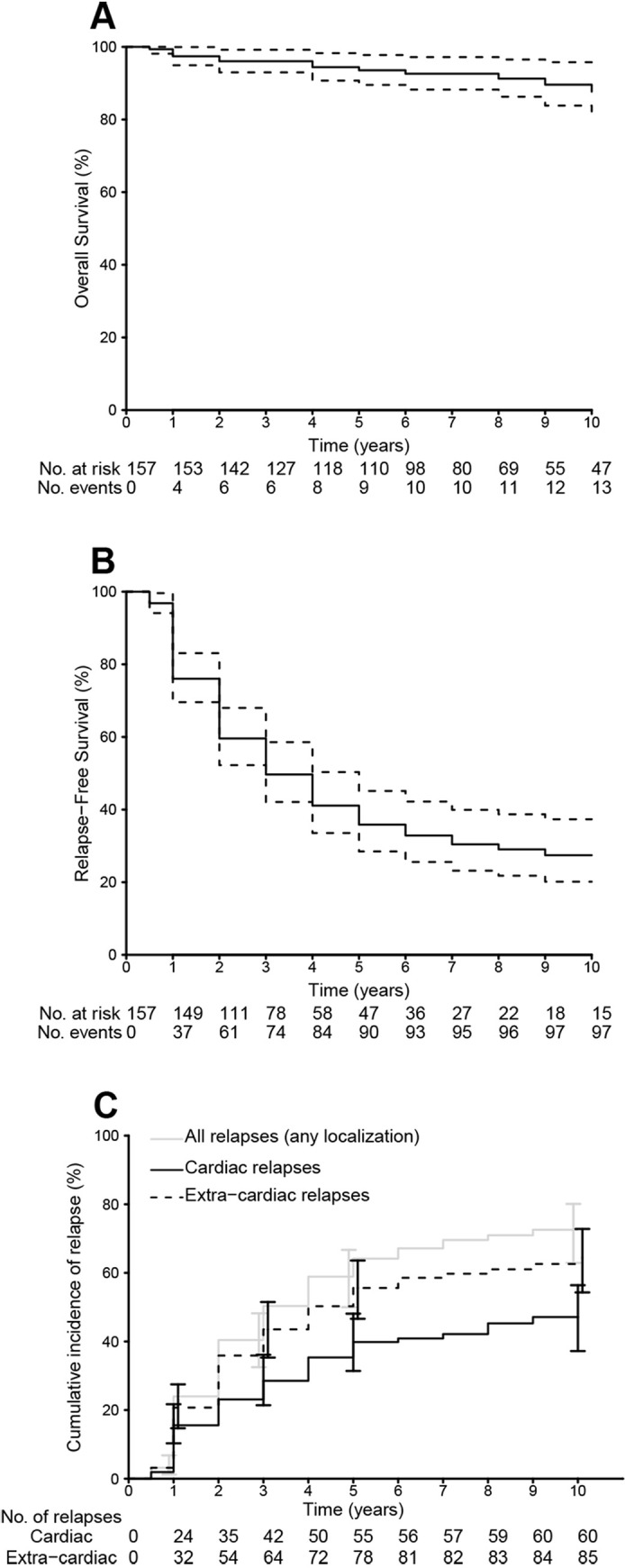
Overall survival of cardiac sarcoidosis patients (Kaplan-Meier) (panel A). Relapse-free survival for all relapses (panel B). Cumulative incidences of cardiac, extra-cardiac, and all relapses (panel C). Overall survival (OS) was defined as the time lapsed from the date of CS diagnosis to the date of death or last follow-up. Relapse-free survival (RFS) was defined as the time lapsed from the date of CS diagnosis to the date of first sarcoidosis relapse, death or last follow-up, whichever occurred first. Both cardiac and non-cardiac relapses were included for RFS analyses.

Thirteen out of 157 patients died during the follow-up. Overall survival rate at 5 and 10 years from CS diagnosis was 93.6% [95% CI, 89.5–97.8] and 89.6% [95% CI, 83.8–95.8], respectively (**Fig 1A**). Deaths were related to CS in four cases, i.e. two cases of refractory cardiac insufficiency, one post-heart transplant, and one unexplained sudden death. The other deaths were due to cerebral events (n = 4), severe asthma (n = 1), lymphoma (n = 1), suicide (n = 1), cardiac surgery not related to CS (n = 1) and unknown cause (n = 1). Univariate analysis found factors associated with fatal outcomes to be older age, LVEF below forty percent, hypertension, abnormal pulmonary function test, and the presence of delayed hypersignal enhancement on cardiac MRI (**Table 4, S3 Table)**.

**Table 4:** Main features associated with overall and relapse-free survivals (all relapses), and cardiac relapses in cardiac sarcoidosis patients (univariate analysis).

		Overall survival			Relapse-free survival			Cardiac relapses†		
Variable		Deaths /patients	HR (95% CI)	P	Relapses/patients	HR (95% CI)	P	Relapses/patients	HR (95% CI)	P
**General features**										
Age at diagnosis (HR per 10 years)		-	1.69 (1.13–2.52)	**0.010**	-	1.11 (0.95–1.29)	0.18	-	1.19 (0.99–1.44)	0.062
Male gender		5/92	0.47 (0.15–1.45)	0.19	57/92	0.92 (0.62–1.36)	0.67	36/92	0.95 (0.57–1.57)	0.83
Ethnic Background										
	Caucasian	8/78	1		45/78	1		52/102	1	
	African/Carib	4/43	0.81 (0.24–2.68)	0.72	36/43	1.78 (1.14–2.78)	**0.011**	22/43	1.47 (0.84–2.59)	0.18
	North African	1/34	0.26 (0.032–2.08)	0.20	20/34	1.17 (0.69–1.99)	0.55	12/34	1.01 (0.51–1.97)	0.99
Smoking		0/20	-	0.19‡	15/20	2.02 (1.16–3.51)	**0.013**	8/20	1.23 (0.58–2.56)	0.59
Hypertension		2/8	4.79 (1.06–21.7)	**0.042**	5/8	2.32 (0.93–5.77)	0.071	4/8	2.33 (0.84–6.47)	0.10
**Extra-cardiac involvement**										
> 2 sites involved		6/90	0.57 (0.19–1.70)	0.31	57/90	0.89 (0.60–1.33)	0.57	32/90	0.66 (0.40–1.37)	0.44
General symptoms		5/67	0.96 (0.31–2.94)	0.94	42/67	1.01 (0.68–1.50)	0.96	23/67	0.82 (0.49–1.37)	0.44
CNS		3/45	0.70 (0.19–2.56)	0.59	31/45	1.43 (0.93–2.18)	0.10	20/45	1.40 (0.82–2.38)	0.22
Lung		13/130		0.17‡	85/130	0.97 (0.53–1.78)	0.93	55/130	1.65 (0.66–4.14)	0.29
Abnormal pulmonary test		8/50	3.27 (1.07–10.0)	**0.038**	34/50	1.20 (0.79–1.82)	0.39	22/50	1.26 (0.75–2.13)	0.38
Eye		1/45	0.21 (0.027–1.61)	0.13	29/45	1.07 (0.69–1.65)	0.76	13/45	0.63 (0.34–1.16)	0.14
Lymph nodes		1/47	0.17 (0.022–1.30)	0.088	29/47	0.91 (0.59–1.41)	0.68	16/47	0.71 (0.40–1.25)	0.24
Skin		6/48	1.95 (0.66–5.81)	0.23	26/48	0.61 (0.39–0.95)	**0.029**	13/48	0.47 (0.25–0.87)	**0.016**
Liver or spleen		3/36	0.88 (0.24–3.22)	0.85	28/36	1.41 (0.91–2.18)	0.13	17/36	1.14 (0.65–1.99)	0.64
Joints		1/37	0.25 (0.033–1.95)	0.19	21/37	0.68 (0.42–1.10)	0.12	11/37	0.58 (0.30–1.11)	0.10
Exocrine glands		2/27	0.84 (0.19–3.80)	0.84	18/27	0.86 (0.51–1.43)	0.56	9/27	0.69 (0.34–1.39)	0.30
ENT		0/8	-	0.34‡	7/8	1.64 (0.76–3.55)	0.21	2/8	0.47 (0.12–1.94)	0.30
Kidney		0/8	-	0.37‡	7/8	4.42 (2.01–9.69)	**0.0002**	6/8	4.10 (1.76–9.58)	**0.001**
**Cardiac involvement**										
NYHA class		2/10	2.80 (0.62–12.6)	0.18	6/10	0.93 (0.40–2.12)	0.86	4/10	1.11 (0.40–3.05)	0.84
Left heart failure		3/15	2.45 (0.67–8.99)	0.18	11/15	1.66 (0.53–2.03)	0.81	10/15	2.01 (1.02–3.95)	**0.044**
Right heart failure		1/3	3.29 (0.42–25.9)	0.26	3/3	1.66 (0.53–5.26)	0.39	3/3	3.29 (1.03–10.5)	**0.045**
AV block		5/27	3.62 (1.18–11.1)	**0.025**	19/27	1.21 (0.72–2.01)	0.47	16/27	2.12 (1.17–3.82)	**0.013**
High degree AV block		4/15	5.56 (1.70–18.2)	**0.005**	13/15	1.80 (0.98–3.30)	0.058	11/15	2.88 (1.45–5.72)	**0.003**
Left bundle branch block		3/10	5.13 (1.41–18.7)	**0.013**	7/10	1.04 (0.48–2.24)	0.92	5/10	1.37 (0.55–3.41)	0.50
LVEF < 40%		3/10	4.88 (1.26–18.9)	**0.022**	9/15	0.87 (0.83–1.73)	0.68	8/15	1.60 (0.75–3.42)	0.22
Septal hypertrophy		1/18	0.59 (0.08–4.51)	0.61	12/18	1.00 (0.54–1.83)	0.99	6/18	0.74 (0.32–1.71)	0.48
Wall motion abnormalities		4/20	2.51 (0.77–8.20)	0.13	16/20	1.18 (0.69–2.01)	0.54	13/20	1.91 (1.03–3.52)	**0.039**
Delayed MRI hypersignal		5/39	2.26 (0.25–20.4)	**0.003**	26/39	1.53 (0.92–2.57)	0.10	19/39	1.86 (0.98–3.52)	**0.056**

Carib, Caribbean; CNS, central nervous system; ENT, ear, nose, throat; NYHA, New York Heart Association; AV, atrio-ventricular; LVEF, left ventricular ejection fraction; MRI, magnetic resonance imaging; ENT, Ear, nose and throat.

‡P-value of Log-Rank test; Estimation of hazards ratio using a Cox regression model was not performed due to the absence of event in one subgroup of interest.

#### Relapses

A hundred and one patients had at least one sarcoidosis-related event, i.e. 63 cardiac relapses and 88 non-cardiac relapses. No death without prior relapse was noted. After 10 years of follow up, the overall RFS rate (cardiac and non-cardiac) was 27.4% (95% CI, 20.2–37.3) (**Fig 1B**). The cardiac RFS rate was 52.9% (95% CI, 44.1–63.4). Cumulative incidences of cardiac and non-cardiac relapses at 1, 3, 5 and 10 years were 6% (95% CI 10–21) and 24% (95% CI 17–30), 32% (21–35) and 50% (43–58), 40% (31–48) and 64% (55–71), and 47% (37–56) and 73% (63–80), respectively (**Fig 1C**).

Univariate analysis showed factors associated with cardiac relapse to be baseline kidney involvement, high degree atrioventricular block, and the presence of late gadolinium enhancement on cardiac MRI (**Table 4, S3 Table)**. The presence of skin involvement was associated with a lower risk of cardiac relapse.

In multivariate analysis, factors associated with cardiac relapse were baseline kidney involvement, left heart failure and wall motion abnormalities on echocardiography, whereas skin involvement was inversely associated.

The impact of immunosuppressive treatments on the relapse risk over a treatment course (any localization or cardiac) is detailed in **Table 5**. Only the administration of intravenous cyclophosphamide was associated with a significant decrease of cardiac relapse risk (HR 0.16, 95% CI 0.03–0.75, *p* = 0.020) compared with the absence of treatment. The HR was 0.37 (0.13–1.08, *p* = 0.069) for the risk of recurrent relapse, including all localization. The administration of glucocorticoids alone, methotrexate or mycophenolic acid were all associated with a non-statistically significant decrease of cardiac relapse rate. Detailed description of treatment sequences included in this analysis is available in **S4 Table**.

**Table 5:** Hazards ratios for relapses (any localization, left; cardiac relapses, right) in cardiac sarcoidosis patients, according to immunosuppressive or immunomodulatory treatments.

Treatment	All relapses /therapeutic sequences	HR (95%CI)‡	P	Cardiac relapses /therapeutic sequences	HR (95%CI)‡	P
**None**	10/13	1	-	8/13	1	-
**Glucocorticoid alone**	17/77	0.51 (0.20–1.31)	0.16	11/77	0.48 (0.18–1.31)	0.15
**Methotrexate**	24/74	1.28 (0.43–3.75)	0.66	9/74	0.62 (0.17–2.19)	0.46
**Mycophenolic acid**	9/54	0.60 (0.21–1.69)	0.33	5/54	0.47 (0.15–1.47)	0.19
**Intravenous cyclophosphamide**	6/48	0.37 (0.13–1.08)	0.069	2/48	0.16 (0.033–0.75)	**0.020**
Other*	9/26	0.76 (0.22–2.61)	0.67	5/26	0.48 (0.16–1.41)	0.18

*Hydroxychloroquine alone (n = 16), infliximab (n = 4), azathioprine alone (n = 3), other immunosuppressant (n = 3)

†Analysis was performed including sequences of treatments between follow-up visits, excluding patients with a clinical therapeutic response as part of their diagnosis Birnie criteria and excluding periods of disease persistence.

‡ Analysis was adjusted on NYHA status (class 3–4 vs. 1–2), presence of cardiac rhythm disorders (yes vs. no), and presence of atrioventricular or ventricular conduction abnormalities (yes vs. no) during follow-up (time-dependent).

## Discussion

In the present study, one of the largest published cohort of patients that has met the new criteria for CS and has had a long follow up, we found that: 1) the 10-year mortality rate was low and associated with older age at CS diagnosis, hypertension, abnormal pulmonary function test, low LVEF, and areas of late gadolinium enhancement on cardiac MRI; 2) the 10-year relapse-rate was high and associated to baseline kidney involvement, left heart failure and the presence wall motion abnormalities on echocardiography; and 3) of the immunosuppressant used, only intravenous cyclophosphamide was associated with a significant decrease in cardiac relapse rates.

Although recent data are reassuring [6, 11, 24], patients with CS have a poorer prognosis than patients without cardiac involvement. The extent of left ventricle dysfunction has been reported as a major predictor of survival [18, 19]. In the study by Chiu et al. at 10 years, all patients with normal ejection fraction were alive whereas patients with severe left ventricular dysfunction had a survival rate of 19% [19]. Some studies found that patients with clinically silent CS have a benign course [25, 30–32]; however contrasting results have been reported [33–36]. In the present series, most deaths were not due to cardiac sarcoidosis, suggesting that CS might be also a marker of an aggressive sarcoidosis. Death was associated with areas of late gadolinium enhancement on cardiac MRI, a sign of myocardium fibrosis/scar reported as a pejorative factor [11, 21–23, 25, 30, 37–39]. Interestingly, repeat FDG PET scan may help to determine the extent of disease activity and to assess the cardiac response to therapy [40–42]. Promising technologies using cardiac FDG PET scan plus MRI might enable concurrent imaging of the two stages of the disease, i.e. inflammation and fibrosis [43].

For patients with extra-pulmonary i.e. cardiac, ocular, neurological, or renal sarcoidosis or hypercalcemia, treatment is recommended [44]. Non-randomized studies have suggested that steroids should be proposed as soon as possible, with good efficacy on ventricular arrhythmia, acute cardiac insufficiency and atrioventricular block [6, 24, 27, 45]. No prognostic difference was found in patients treated with high or moderate doses of prednisone [46]. Immunosuppressant, often used in refractory cases and/or if steroid side effects, included methotrexate [6, 22, 24, 25], azathioprine [22, 47], cyclophosphamide [6, 48], mycophenolate mofetil [22, 24] and more recently infliximab [49–53]. In the present study, only intravenous cyclophosphamide was associated with a significant decrease in cardiac relapse rates. Other immunosuppressive drugs used were also associated with a lower cardiac relapse risk (i.e. glucocorticoid alone, methotrexate or mycophenolic acid). Probably due to the lack of sufficient power and/or insufficient efficacy and/or use as second-line therapies in refractory CS, the latter results were not statistically significant. In the present series, the number of patients who received infliximab was too small to draw firm conclusions [49–53]. Of note, analyses on treatments should be interpreted with caution as treatments were not randomised (possible confounding factors), and sample sizes of some treatment were small (under power). Despite a widespread use of steroids and immunosuppressant drugs, the adverse effect rate remained low. This is probably related to the low dose of steroids patients received at the end of follow up. This highlights a benefit/risk balance in favour of long-term immunosuppression in CS patients, particularly if patients show factors predictive of poor outcome or cardiac relapse.

## Limitations

Due to the rarity of the disease, we analysed retrospective data. A referral centre bias may explain some of the characteristics of our cohort (multi-systemic severe forms of sarcoidosis, rarity of atrio-ventricular block). The clinical variety of CS required the use of complex statistical models. A multivariate analysis was not feasible for overall survival due to the small number of events. Due to the long enrollment period, we cannot exclude possible implications of change in backward cardiovascular therapies or diagnostic tools on outcomes. Only a part of the patients did cardiac MRI and cardiac FDG-PET scan, both fundamental in relapse and prognostic evaluation. Also, as mentioned above, immunosuppressive treatments were not randomised.

## Conclusion

In patients with cardiac sarcoidosis, more frequent relapses were found to be associated with baseline kidney involvement, left heart failure and the presence of wall motion abnormalities on echocardiography. Mortality rate was low and associated to older age, arterial hypertension, abnormal pulmonary function tests, low LVEF and the presence of areas of late gadolinium enhancement on cardiac MRI. Immunosuppressive therapy with intravenous cyclophosphamide is associated with lower relapse rates and might be especially of interest when predictive factors of poor outcome or relapses are present. Such results should be confirmed in randomized controlled trials.

## Supporting information

S1 Table NYHA class of dyspnea at baseline and during the follow up, according to baseline pulmonary function tests. (DOCX)

S2 Table Associations of immuno-suppressive or immuno-modulatory treatments in the entire database [associations are grouped according to the main active molecule received (bold characters)]. (DOCX)

S3 Table Univariate analyses of corresponding Main Table 1 (S1), Table 2 (S2) and Table 3 (S3). (DOCX)

S4 Table Detailed description of sequences of treatment (associations of immuno-suppressive or immuno-modulatory treatments), included in the analysis of the association of treatment with recurrent relapses. (DOCX)
