# Supplementary material for: Cardiac sarcoidosis: A long term follow up study
Source: PLoS One. 2020 Sep 18;15(9):e0238391. doi: 10.1371/journal.pone.0238391 (PMC7500618; doi:10.1371/journal.pone.0238391)
Supplement: S1 Table — (DOCX) [file pone.0238391.s001.docx]

**Supplementary Table 1.** NYHA class of dyspnea at baseline and during the follow up, according to baseline pulmonary function tests.

| **Variables** | **Total** | **Normal baseline pulmonary function tests** | **Abnormal baseline pulmonary function tests** |
| --- | --- | --- | --- |
| Number of patients | 157 | 104 | 50 |
| NYHA class of dyspnea, n (%) |  |  |  |
| 1 | 119 (76) | 82 (79) | 35 (70) |
| 2 | 28 (18) | 17 (16) | 11 (22) |
| 3 | 7 (4) | 2 (2) | 4 (8) |
| 4 | 3 (2) | 3 (3) | 0 (0) |
|  |  |  |  |

NYHA, New York Heart Association
