# Supplementary material for: Cardiac sarcoidosis: A long term follow up study
Source: PLoS One. 2020 Sep 18;15(9):e0238391. doi: 10.1371/journal.pone.0238391 (PMC7500618; doi:10.1371/journal.pone.0238391)
Supplement: S2 Table — (DOCX) [file pone.0238391.s002.docx]

**Supplementary Table 2:** Associations of immuno-suppressive or immuno-modulatory treatments in the entire database [associations are grouped according to the main active molecule received (bold characters)].

| **Treatment associations** | **No. of patients (%)*** |
| --- | --- |
| Total number of patients with follow-up | 157 |
| **No treatment** | **38 (24)** |
| **Glucocorticoids only** | **92 (59)** |
| **Methotrexate** | **59 (38) †** |
| GC + MTX | 54 (35) |
| GC + MTX + HCQ | 5 (3) |
| GC + MTX + MMF | 1 (<1) |
| GC + MTX + other IS | 1 (<1) |
| GC + MTX + CYC | 2 (1) |
| **Mycophenolic acid** | **45 (29) †** |
| GC + MMF | 41 (26) |
| GC + MMF + HCQ | 3 (2) |
| GC + MMF + other IS | 1 (<1) |
| **Cyclophosphamide** | **79 (51) †** |
| GC + CYC | 74 (47) |
| GC + CYC + HCQ | 4 (3) |
| GC + CYC + HCQ + other IS | 1 (<1) |
| CYC | 1 (<1) |
| GC + CYC + other IS | 1 (<1) |
| GC + CYC + MMF | 1 (<1) |
| **Hydroxychloroquine** | **29 (19) †** |
| GC + HCQ | 18 (12) |
| HCQ | 5 (3) |
| GC + HCQ + other IS | 1 (<1) |
| GC + AZA + HCQ | 2 (1) |
| **IFX** | **14 (9) †** |
| GC + MTX + IFX | 7 (4) |
| GC + IFX | 6 (4) |
| GC + IFX + MMF | 1 (<1) |
| GC + MTX + IFX + HCQ | 2 (1) |
| GC + IFX + AZA + HCQ | 1 (<1) |
| MTX + IFX + HCQ | 1 (<1) |
| **Other** | **10 (6) †** |
| GC + AZA | 8 (5) |
| GC + other IS | 2 (1) |

*: Number of patients who received the molecule (bold character) or specific association at least once during the follow-up (i.e. for at least one follow-up sequence, between two visits)

†: Some patients received different associations among the main groups; detailed association counts (%) within groups (MTX, MMF, CYC, HCQ, IFS, other) may add up to above 100%.

GC, glucocorticoids; MTX, methotrexate; MMF, mycophenolic acid; CYC, IV cyclophosphamide; HCQ, hydroxychloroquine; IFX, infliximab; IS, immunosuppressant; AZA, azathioprine.
