# Supplementary material for: Cardiac sarcoidosis: A long term follow up study
Source: PLoS One. 2020 Sep 18;15(9):e0238391. doi: 10.1371/journal.pone.0238391 (PMC7500618; doi:10.1371/journal.pone.0238391)
Supplement: S3 Table — (DOCX) [file pone.0238391.s003.docx]

**Supplementary Table 3:** Univariate analyses of corresponding Main Table 1 (S1), Table 2 (S2) and Table 3 (S3).

***S1- Main extra-cardiac features***

|  | | **Overall survival** | | | **Relapse-free survival** | | | **Cardiac relapses**† | | |
| --- | --- | --- | --- | --- | --- | --- | --- | --- | --- | --- |
| **Variable** | | **Deaths /patients** | **HR (95% CI)** | **P** | **Relapses/patients** | **HR (95% CI)** | **P** | **Relapses/patients** | **HR (95% CI)** | **P** |
| **General features** | | | | | | | | | | |
| Age at diagnosis (HR per 10 years) | | - | 1.69 (1.13-2.52) | **0.010** | - | 1.11 (0.95-1.29) | 0.18 | - | 1.19 (0.99-1.44) | 0.062 |
| Male gender | | 5/92 | 0.47 (0.15-1.45) | 0.19 | 57/92 | 0.92 (0.62-1.36) | 0.67 | 36/92 | 0.95 (0.57-1.57) | 0.83 |
| Ethnic Background | |  | | |  | | |  | | |
|  | Caucasian | 8/78 | 1 |  | 45/78 | 1 |  | 52/102 | 1 |  |
|  | African/Carib | 4/43 | 0.81 (0.24-2.68) | 0.72 | 36/43 | 1.78 (1.14-2.78) | **0.011** | 22/43 | 1.47 (0.84-2.59) | 0.18 |
|  | North African | 1/34 | 0.26 (0.032-2.08) | 0.20 | 20/34 | 1.17 (0.69-1.99) | 0.55 | 12/34 | 1.01 (0.51-1.97) | 0.99 |
| Smoking | | 0/20 | - | 0.19‡ | 15/20 | 2.02 (1.16-3.51) | **0.013** | 8/20 | 1.23 (0.58-2.56) | 0.59 |
| Hypertension | | 2/8 | 4.79 (1.06-21.7) | **0.042** | 5/8 | 2.32 (0.93-5.77) | 0.071 | 4/8 | 2.33 (0.84-6.47) | 0.10 |
| **Extra-cardiac involvement** | | | | | | | | | | |
| > 2 sites involved | | 6/90 | 0.57 (0.19-1.70) | 0.31 | 57/90 | 0.89 (0.60-1.33) | 0.57 | 32/90 | 0.66 (0.40-1.37) | 0.44 |
| Pulmonary classification ( Chest X-ray) | |  |  | 0.48‡ |  |  |  |  |  |  |
| Class 0 | | 0/16 | - |  | 12/16 | 1 |  | 5/16 | 1 |  |
| Class I | | 3/38 | - |  | 23/38 | 0.83 (0.41-1.67) | 0.60 | 17/38 | 1.68 (0.62-4.56) | 0.31 |
| Class II | | 8/67 | - |  | 45/67 | 1.04 (0.55-1.98) | 0.89 | 29/67 | 1.76 (0.68-4.57) | 0.25 |
| Class III | | 2/25 | - |  | 17/25 | 1.03 (0.49- 2.17) | 0.93 | 9/25 | 1.34 (0.45-4.02) | 0.60 |
| General symptoms | | 5/67 | 0.96 (0.31-2.94) | 0.94 | 42/67 | 1.01 (0.68-1.50) | 0.96 | 23/67 | 0.82 (0.49-1.37) | 0.44 |
| Skin | | 6/48 | 1.95 (0.66-5.81) | 0.23 | 26/48 | 0.61 (0.39-0.95) | **0.029** | 13/48 | 0.47 (0.25-0.87) | **0.016** |
| Lymph nodes | | 1/47 | 0.17 (0.022-1.30) | 0.088 | 29/47 | 0.91 (0.59-1.41) | 0.68 | 16/47 | 0.71 (0.40-1.25) | 0.24 |
| CNS | | 3/45 | 0.70 (0.19-2.56) | 0.59 | 31/45 | 1.43 (0.93-2.18) | 0.10 | 20/45 | 1.40 (0.82-2.38) | 0.22 |
| Eye | | 1/45 | 0.21 (0.027-1.61) | 0.13 | 29/45 | 1.07 (0.69-1.65) | 0.76 | 13/45 | 0.63 (0.34-1.16) | 0.14 |
| Joints | | 1/37 | 0.25 (0.033-1.95) | 0.19 | 21/37 | 0.68 (0.42-1.10) | 0.12 | 11/37 | 0.58 (0.30-1.11) | 0.10 |
| Liver or spleen | | 3/36 | 0.88 (0.24-3.22) | 0.85 | 28/36 | 1.41 (0.91-2.18) | 0.13 | 17/36 | 1.14 (0.65-1.99) | 0.64 |
| Exocrine glands | | 2/27 | 0.84 (0.19-3.80) | 0.84 | 18/27 | 0.86 (0.51-1.43) | 0.56 | 9/27 | 0.69 (0.34-1.39) | 0.30 |
| ENT | | 0/8 | - | 0.34‡ | 7/8 | 1.64 (0.76-3.55) | 0.21 | 2/8 | 0.47 (0.12-1.94) | 0.30 |
| Kidney | | 0/8 | - | 0.37‡ | 7/8 | 4.42 (2.01-9.69) | **0.0002** | 6/8 | 4.10 (1.76-9.58) | **0.001** |
| Peripheral nervous system | | 1/5 | 2.25 (0.29-17.4) | 0.44 | 5/5 | 1.70 (0.69-4.18) | 0.25 | 3/5 | 1.28 (0.40 to 4.10) | 0.68 |
| Bones | | 0/4 | - | 0.48‡ | 2/4 | 0.62 (0.15-2.51) | 0.50 | 2/4 | 1.28 (0.31-5.26) | 0.73 |
| Digestive tract | | 0/3 | - | 0.61‡ | 2/3 | 1.23 (0.30-5.01) | 0.77 | 2/3 | 2.45 (0.60-10.1) | 0.21 |

*‡P-value of Log-Rank test; Estimation of hazards ratio using a Cox regression model was not performed due to the absence of event in one subgroup of interest*

***S2 – Main clinical cardiac features***

|  | **Overall survival** | | | **Relapse-free survival** | | | **Cardiac relapses**† | | |
| --- | --- | --- | --- | --- | --- | --- | --- | --- | --- |
| **Variable** | **Deaths /patients** | **HR (95% CI)** | **P** | **Relapses/patients** | **HR (95% CI)** | **P** | **Relapses/patients** | **HR (95% CI)** | **P** |
| **Cardiac involvement** | | | | | | | | | |
| Palpitation | 1/20 | 0.50 (0.06-3.84) | 0.50 | 12/20 | 0.79 (0.43-1.44) | 0.44 | 8/20 | 0.94 (0.45-1.97) | 0.87 |
| Syncope | 1/10 | 1.68 (0.22-13.1) | 0.62 | 6/10 | 0.88 (0.38-2.00) | 0.75 | 4/10 | 1.07 (0.39-2.96) | 0.89 |
| NYHA class | 2/10 | 2.80 (0.62-12.6) | 0.18 | 6/10 | 0.93 (0.40-2.12) | 0.86 | 4/10 | 1.11 (0.40-3.05) | 0.84 |
| Left heart failure | 3/15 | 2.45 (0.67-8.99) | 0.18 | 11/15 | 1.66 (0.53-2.03) | 0.81 | 10/15 | 2.01 (1.02-3.95) | **0.044** |
| Right heart failure | 1/3 | 3.29 (0.42-25.9) | 0.26 | 3/3 | 1.66 (0.53-5.26) | 0.39 | 3/3 | 3.29 (1.03-10.5) | **0.045** |
| Atrial troubles | 3/55 | 0.51 (0.14-1.84) | 0.30 | 35/55 | 0.99 (0.66-1.50) | 0.98 | 20/55 | 0.86 (0.51-1.47) | 0.58 |
| Sinusal tachycardia | 2/49 | 0.40 (0.09-1.81) | 0.24 | 32/49 | 1.17 (0.76-1.78) | 0.47 | 17/49 | 0.85 (0.49-1.49) | 0.58 |
| Fibrillation or flutter | 1/9 | 1.02 (0.13-7.95) | 0.98 | 5/9 | 0.57 (0.23-1.41) | 0.23 | 3/9 | 0.66 (0.21-2.10) | 0.48 |
| Ventricular arrhythmia | 1/27 | 0.41 (0.05-3.17) | 0.39 | 16/27 | 0.69 (0.40-1.20) | 0.19 | 9/27 | 0.66 (0.31-1.39) | 0.27 |
| Ventricular extrasystoles | 0/21 | - | 0.16*‡* | 13/21 | 0.73 (0.40-1.34) | 0.31 | 8/21 | 0.78 (0.36-1.72) | 0.54 |
| Ventricular tachycardia | 1/13 | 0.82 (0.11-6.35) | 0.85 | 8/13 | 0.54 (0.25-1.17) | 0.12 | 4/13 | 0.42 (0.13-1.35) | 0.15 |
| AV block | 5/27 | 3.62 (1.18-11.1) | 0.025 | 19/27 | 1.21 (0.72-2.01) | 0.47 | 16/27 | 2.12 (1.17-3.82) | **0.013** |
| High degree AV block | 4/15 | 5.56 (1.70-18.2) | 0.005 | 13/15 | 1.80 (0.98-3.30) | 0.058 | 11/15 | 2.88 (1.45-5.72) | **0.003** |
| Ventricular block | 4/38 | 1.46 (0.45-4.75) | 0.53 | 22/38 | 0.67 (0.42-1.07) | 0.095 | 13/38 | 0.75 (0.40-1.38) | 0.35 |
| Left bundle branch block | 3/10 | 5.13 (1.41-18.7) | 0.013 | 7/10 | 1.04 (0.48-2.24) | 0.92 | 5/10 | 1.37 (0.55-3.41) | 0.50 |
| Q wave/ST-T changes | 3/27 | 1.36 (0.37-4.94) | 0.64 | 21/27 | 1.28 (0.79-2.07) | 0.32 | 14/27 | 1.37 (0.76-2.49) | 0.30 |
| LVEF < 40% | 3/10 | 4.88 (1.26-18.9) | **0.022** | 9/15 | 0.87 (0.83-1.73) | 0.68 | 8/15 | 1.60 (0.75-3.42) | 0.22 |
| Septal hypertrophy | 1/18 | 0.59 (0.08-4.51) | 0.61 | 12/18 | 1.00 (0.54-1.83) | 0.99 | 6/18 | 0.74 (0.32-1.71) | 0.48 |
| Delayed MRI hypersignal | 5/39 | 2.26 (0.25-20.4) | **0.003** | 26/39 | 1.53 (0.92-2.57) | 0.10 | 19/39 | 1.86 (0.98-3.52) | **0.056** |

*‡P-value of Log-Rank test; Estimation of hazards ratio using a Cox regression model was not performed due to the absence of event in one subgroup of interest*

***S3 – Main imaging cardiac features***

|  | **Overall survival** | | | **Relapse-free survival** | | | **Cardiac relapses**† | | |
| --- | --- | --- | --- | --- | --- | --- | --- | --- | --- |
| **Variable** | **Deaths /patients** | **HR (95% CI)** | **P** | **Relapses/patients** | **HR (95% CI)** | **P** | **Relapses/patients** | **HR (95% CI)** | **P** |
| **Echocardiography** | | | | | | | | | |
| Diffuse hypokinesia | 6/41 | 2.47 (0.75-8.10) | 0.14 | 29/41 | 1.09 (0.69-1.71) | 0.71 | 23/41 | 1.74 (1.04-2.93) | **0.037** |
| Localized hypokinesia | 3/40 | 0.70 (0.19-2.55) | 0.58 | 29/40 | 1.08 (0.70-1.67) | 0.72 | 20/40 | 1.30 (0.76-2.22) | 0.33 |
| Wall motion abnormalities | 4/20 | 2.51 (0.77-8.20) | 0.13 | 16/20 | 1.18 (0.69-2.01) | 0.54 | 13/20 | 1.91 (1.03-3.52) | **0.039** |
| Thick interventricular septum | 1/18 | 0.59 (0.08-4.51) | 0.61 | 12/18 | 1.00 (0.54-1.83) | 0.99 | 6/18 | 0.74 (0.32-1.71) | 0.48 |
| Abnormal pericardium | 1/18 | 0.56 (0.07-4.35) | 0.58 | 11/18 | 1.01 (0.54-1.90) | 0.97 | 6/18 | 0.76 (0.33-1.77) | 0.52 |
| Left ventricular ejection fraction |  |  |  |  |  |  |  |  |  |
| > 50 % | 7/104 | 1 |  | 63/104 | 1 |  | 42/104 | 1 | 0.14 |
| 50-40 % | 1/17 | 1.01 (0.12-8.24) | 0.99 | 13/17 | 1.26 (0.68-2.34) | 0.46 | 7/17 | 0.89 (0.38-2.10) | 0.79 |
| < 40 % | 3/10 | 4.88 (1.26-18.9) | 0.022 | 5/10 | 0.84 (0.34-2.10) | 0.71 | 5/10 | 1.53 (0.61-3.88) | 0.37 |
| **Scintigraphy (n=91)** | | | | | | | | | |
| Hypersignals (T1 mapping)* | 3/24 | 3.94 (0.66-23.6) | 0.13 | 17/24 | 1.28 (0.73-2.24) | 0.39 | 11/24 | 1.25 (0.62-2.53) | 0.54 |
| Early gadolinium enhancement † | 1/12 | 2.26 (0.25-20.4) | 0.47 | 6/12 | 0.94 (0.40-2.17) | 0.88 | 6/12 | 2.02 (0.84-4.86) | 0.12 |
| Delayed MRI hypersignal | 5/39 | - | **0.003***‡* | 26/39 | 1.53 (0.92-2.57) | 0.10 | 19/39 | 1.86 (0.98-3.52) | **0.056** |
| Localized hypokinesia† | 1/9 | 3.04 (0.34-27.4) | 0.32 | 5/9 | 0.69 (0.27-1.71) | 0.42 | 3/9 | 0.89 (0.27-2.89) | 0.84 |
| Low left ventricular ejection fraction† | 2/28 | 1.87 (0.31-11.3) | 0.49 | 19/28 | 1.15 (0.67-1.97) | 0.62 | 11/28 | 1.07 (0.53-2.17) | 0.85 |
| Abnormal pericardium** | 0/9 | - | 0.45*‡* | 3/9 | 0.46 (0.14-1.48) | 0.19 | 1/9 | 0.25 (0.034-1.84) | 0.17 |
| Cardiac PET Scan (n=37) |  |  |  |  |  |  |  |  |  |
| Patchy uptake | 0/12 | - | **-** | 6/12 | 1.42 (0.55-3.67) | 0.47 | 2/12 | 0.51 (0.11-2.28) | 0.37 |

**n=87; †: n=88; **n=8 ; ‡P-value of Log-Rank test; Estimation of hazards ratio using a Cox regression model was not performed due to the absence of event in one subgroup of interest*
