# Supplementary material for: Cardiac sarcoidosis: A long term follow up study
Source: PLoS One. 2020 Sep 18;15(9):e0238391. doi: 10.1371/journal.pone.0238391 (PMC7500618; doi:10.1371/journal.pone.0238391)
Supplement: S4 Table — (DOCX) [file pone.0238391.s004.docx]

**Supplementary Table 4:** Detailed description of sequences of treatment (associations of immuno-suppressive or immuno-modulatory treatments), included in the analysis of the association of treatment with recurrent relapses (Main Table 5). Association were grouped according to the main active molecule for the analysis reported in main table 5.

| **Treatment association** | **No. of sequences (%)** |
| --- | --- |
| **No treatment** | 13 (4) |
| **Glucocorticoids alone** | 77 (26) |
| **Methotrexate** | 74 (25) |
| GC + MTX | 61 (21) |
| GC + MTX + HCQ | 4 (1) |
| GC + MTX + MMF | 4 (1) |
| GC + MTX + other IS | 3 (1) |
| GC + MTX + CYC | 2 (1) |
| **Mycophenolic acid** | 54 (18) |
| GC + MMF | 44 (15) |
| GC + MMF + other IS | 6 (2) |
| GC + MMF + HCQ | 4 (1) |
| **Cyclophosphamide** | 48 (16) |
| GC + CYC | 44 (15) |
| GC + CYC + HCQ | 2 (1) |
| GC + CYC + other IS | 1 (0) |
| CYC | 1 (0) |
| **Hydroxychloroquine** | 16 (5) |
| GC + HCQ | 11 (4) |
| HCQ | 4 (1) |
| GC + AZA + HCQ | 1 (0) |
| **Infliximab** | 4 (1) |
| GC + IFX | 1 (0) |
| GC + IFX + AZA + HCQ | 1 (0) |
| GC + MTX + IFX + HCQ | 1 (0) |
| MTX + IFX + HCQ | 1 (0) |
| **Other** | 6 (2) |
| GC + other IS | 3 (1) |
| GC + AZA | 3 (1) |

GC, glucocorticoid; MTX, methotrexate; MMF, mycophenolic acid; CYC, iv cyclophosphamide; HCQ, hydroxychloroquine; IFX, infliximab; IS, immunosuppressant; AZA, azathioprine.
